# Domestic

**Published:** 1841-09-04

**Authors:** 


### MEDICAL EXAMINER.

DEVOTED TO MEDICINE, SURGERY, AND THE COLLATERAL SCIENCES.

No. 36.] PHILADELPHIA, SATURDAY, SEPTEMBER 4, 1841. [Vol. IV.

### DOMESTIC.

### HEALTH OF THE CITY

Interments *in the City and Liberties of Philadelphia, from the 21st to the 28th of August.*

| Diseases. | Adults. | Children. | Diseases. | Adults. | Children. |
|---|---|---|---|---|---|
| Abscess of Liver, | 1 | 0 | *Brought forward,* | 35 | 42 |
| Aneurism of | | | Marasmus, | 0 | 4 |
| Heart, | 0 | 1 | Malformation, | 0 | 2 |
| Apoplexy, | 2 | 0 | Old age, | 2 | 0 |
| Croup, | 0 | 1 | Palsy, | 1 | 0 |
| Consumption of | | | Scrofula, | 2 | 0 |
| the lungs, | 12 | 6 | Small pox, | 0 | 5 |
| Convulsions, | 0 | 4 | Still-born, | 0 | 6 |
| Diarrhœa, | 2 | 6 | Suicide, | 2 | 0 |
| Dropsy, | 1 | 0 | Summer Com- | | |
| —— of the head, | 0 | 5 | plaint, | 0 | 18 |
| Disease of heart, | 1 | 0 | Spitting of blood, | 1 | 0 |
| —— Hip Joint, | 0 | 1 | Teething, | 0 | 1 |
| —— Prostate | | | Unknown, | 0 | 5 |
| gland, | 1 | 0 | | — | — |
| Drowned, | 2 | 1 | Total, 126— | 43 | 83 |
| Dysentery, | 6 | 2 | | | |
| Debility | 1 | 4 | —— | | |
| Effusion on brain, | 0 | 1 | | | |
| Fever, | 0 | 1 | Of the above, there | | |
| —— Congestive, | 1 | 0 | were under 1 year, | | 47 |
| —— Remittent, | 1 | 0 | From 1 to 2 | | 18 |
| —— Bilious, | 1 | 0 | 2 to 5 | | 8 |
| —— Scarlet, | 0 | 1 | 5 to 10 | | 4 |
| Inflammation of | | | 10 to 15 | | 1 |
| the Brain, | 0 | 1 | 15 to 20 | | 5 |
| —— Lungs, | 0 | 1 | 20 to 30 | | 7 |
| —— Stomach and | | | 30 to 40 | | 12 |
| Bowels, | 0 | 1 | 40 to 50 | | 8 |
| —— Bowels, | 1 | 4 | 50 to 60 | | 7 |
| —— Peritonæum, | 1 | 0 | 60 to 70 | | 4 |
| Intemperance, | 1 | 0 | 70 to 80 | | 4 |
| Jaundice, | 0 | 1 | 80 to 90 | | 1 |
| | — | — | | | —— |
| *Carried forward,* | 35 | 42 | Total, | | 126 |

Of the above there were 6 from the almshouse, 9 people of colour, and 1 from the country, which are included in the total amount.

